# SNP HiTLink: a high-throughput linkage analysis system employing dense SNP data

**DOI:** 10.1186/1471-2105-10-121

**Published:** 2009-04-24

**Authors:** Yoko Fukuda, Yasuo Nakahara, Hidetoshi Date, Yuji Takahashi, Jun Goto, Akinori Miyashita, Ryozo Kuwano, Hiroki Adachi, Eiji Nakamura, Shoji Tsuji

**Affiliations:** 1 Department of Neurology, Graduate School of Medicine, the University of Tokyo, Tokyo 113-8655, Japan; 2 Department of Clinical Genomics, the University of Tokyo Hospital, Tokyo 113-8655, Japan; 3 Department of Molecular Genetics, Bioresource Science Branch, Center for Bioresources, Brain Research Institute, Niigata University, Niigata 951-8585, Japan; 4 Gene Diversity Software Department, Dynacom Co Ltd., Kanagawa 220-0003, Japan

## Abstract

**Background:**

During this recent decade, microarray-based single nucleotide polymorphism (SNP) data are becoming more widely used as markers for linkage analysis in the identification of loci for disease-associated genes. Although microarray-based SNP analyses have markedly reduced genotyping time and cost compared with microsatellite-based analyses, applying these enormous data to linkage analysis programs is a time-consuming step, thus, necessitating a high-throughput platform.

**Results:**

We have developed SNP HiTLink (SNP High Throughput Linkage analysis system). In this system, SNP chip data of the Affymetrix Mapping 100 k/500 k array set and Genome-Wide Human SNP array 5.0/6.0 can be directly imported and passed to parametric or model-free linkage analysis programs; MLINK, Superlink, Merlin and Allegro. Various marker-selecting functions are implemented to avoid the effect of typing-error data, markers in linkage equilibrium or to select informative data.

**Conclusion:**

The results using the 100 k SNP dataset were comparable or even superior to those obtained from analyses using microsatellite markers in terms of LOD scores obtained. General personal computers are sufficient to execute the process, as runtime for whole-genome analysis was less than a few hours. This system can be widely applied to linkage analysis using microarray-based SNP data and with which one can expect high-throughput and reliable linkage analysis.

## Background

Recent technological development of high-density SNP chips has made it practical to genotype more than a million SNPs. Because microarray-based dense SNP typing requires less time and typing cost and can provide much more information than PCR-based microsatellite markers, it is now widely recognized as a powerful tool for linkage analysis [1-3]. To apply SNP information to genome-wide high-throughput linkage analysis, however, there are some difficulties as follows. 1) LINKAGE file preparation: Most linkage analysis software accepts LINKAGE format genotype data containing information on each marker for pairwise analysis or that on all markers on each chromosome for multipoint analysis. For example, pairwise analysis of 1000 SNPs on a chromosome using MLINK [4,5], a pairwise linkage analysis program, means preparing 1000 genotype files and 1000 marker information files, followed by running the program 1000 times. In multipoint analysis, information on the 1000 genotypes or marker information containing intermarker distances should be described in one file. Preparation of these files based on the information contained in the CHP file, which is generated by Affymetrix Genotyping Console ™ from firstly created CEL files in genotyping assays, are laborious and time-consuming for researchers. 2) Typing error: In microarray-based SNP detection, typing error is rare but inevitable because several factors such as the quality of genomic DNA, experimental conditions and the number of samples incorporated in the clustering of genotypes, can lead to inaccurate SNP calling [6-9]. This relatively rare miscalling, however, can lead to critical miscalculation in linkage analysis, particularly when parent genotypes are lacking, or in multipoint analysis. Therefore, estimation and elimination of typing error data would be necessary for reliable results. 3) Linkage disequilibrium (LD) in neighboring markers for multipoint analysis: In algorithms of multipoint linkage analysis, it is usually assumed that all markers are in linkage equilibrium with each other. Markers in LD should be appropriately eliminated to avoid inaccurate calculation, which can be accompanied by inflation of LOD scores [10,11]. This is particularly important when using recently developed high-density SNP chips.

We have herein developed SNP HiTLink that directly accepts Affymetrix SNP CHP files and perform parametric/nonparametric linkage analyses with quite flexible marker selection functionalities.

## Implementation

SNP HiTLink works under Windows XP SP2 or later/Vista (Use only 32-bit versions of Windows) and unix (supporting perl 5) OS [Additional files 1 and 2]. MLINK (LINKAGE/fastlink), Superlink, Merlin and Allegro should be installed in Unix OS. MLINK is included in FASTLINK package. Allegro is available from deCODE genetics, Inc. At present, SNP HiTLink accepts files in the CHP file format (filename.chp) of the Affymetrix Mapping 100 k/500 k array set and Genome-Wide Human SNP array 5.0/6.0. SNP HiTLink consists of two processes. The first process creates necessary data files by the program described in the Visual Basic programming on Windows OS, and these files are then transferred to Unix OS. The Perl script files invoke necessary linkage programs with necessary data files on Unix OS.

**Figure 1:**
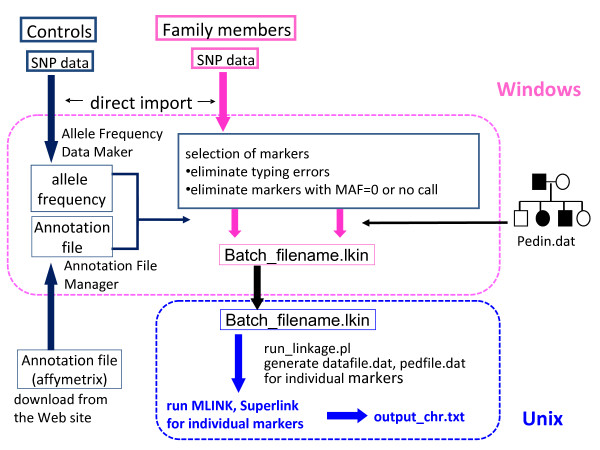
**Flowcharts of data processing for pair-wise linkage analysis employing MLINK or Superlink by SNP HiTLink**. In Windows OS, import of SNP data, generation of allele frequency file, annotation file and lkin file are conducted along with selection of markers. After lkin file is transported to Unix OS, run_linkage.pl carries out continuous run of MLINK or Superlink by rewriting pedin.pre and pedin.dat files for each marker.

**Figure 2:**
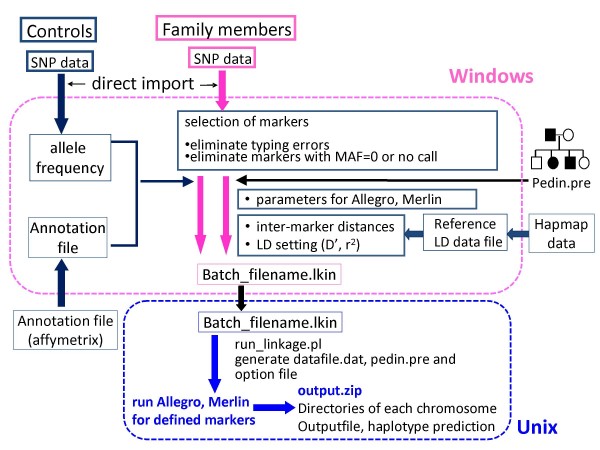
**Flowcharts of data processing for multipoint linkage analysis employing Merlin or Allegro with SNP HiTLink**. Procedures are basically similar to those by pair-wise analysis except that the model setting, selection of intermarker distances are executable here. run_linkage.pl carries out a run of Allegro or Merlin with all selected markers by writing whole information in pedin.pre, datain.dat.

Figures 1 and 2 shows a flow-chart representing the process of linkage analysis. "Allele Frequency Data Maker" and "Annotation File Manager" programs are implemented in SNP HiTLink to obtain allele frequencies and SNP information. These are automatically generated from CHP files of control samples and annotation files downloaded from the Affymetrix web page. When analyzing a new family, users need to prepare a "map" file and "pedin.dat" (MLINK, Superlink) or "pedin.pre" (Merlin, Allegro) files manually by a text editor [see Additional file 3]. Although "pedin.dat" or "pedin.pre" should be described basically in the standard LINKAGE format (see manuals of each program for detail), no genotype data are required here. "map" files link an individual number described in "pedin.dat" or "pedin.pre" to the name of a "filename.chp" file from each individual.

**Figure 3:**
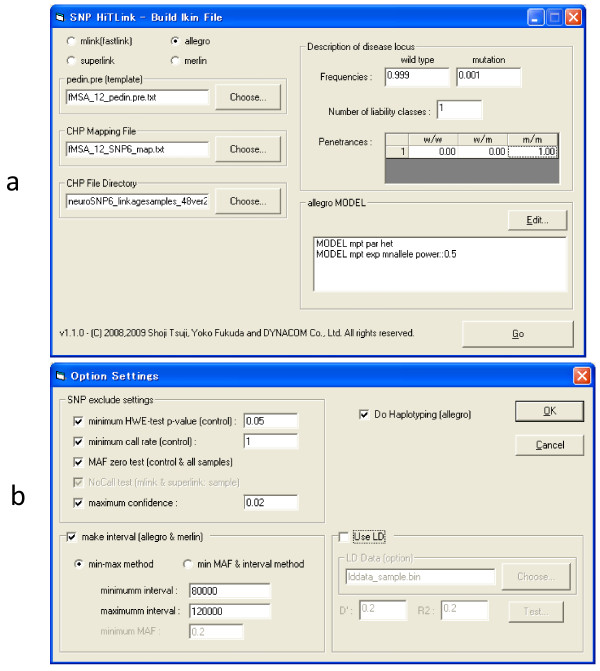
**Interface of first step of "build lkin file"(a) and "option settings"(b) of SNP HiTLink**.

SNP HiTLink can run four standard linkage analysis programs, MLINK [4,5], Superlink [12], Merlin [13] and Allegro [14,15]. Pair-wised analysis is supported by MLINK, Superlink and Allegro while multipoint analysis can be conducted by Merlin and Allegro in SNP HiTLink. Figure 3 shows the interface of the first step of the "build lkin file" (Figure 3a) and "option settings" (Figure 3b). For the pairwise linkage analysis by MLINK or Superlink, the user chooses pedin.dat and map files then specify the directory containing the CHP files. Disease gene frequency and liability class are defined here. For performing Merlin or Allegro, the user chooses pedin.pre files instead of pedin.dat, and then chooses model options that are identical to those originally implemented in Merlin and Allegro. After selecting programs and models, the user sets the marker-selecting options in which we implemented various parameters to eliminate typing errors and uninformative markers classified as follows.

1) To eliminate markers with typing errors, HWE, call rate, and confidence score are used as the effective indexes because deviations from HWE, lower call rates and higher confidence scores at particular markers sometimes suggest problems with genotyping. 2) To select informative markers useful for linkage analyses, the 'MAF zero test' and 'No call test' will be performed because these markers are totally uninformative. 3) To avoid employing markers in LD in the multipoint analysis, appropriate intermarker distances or D' and r^2^, which are indexes of LD, can be defined by users.

•  HWE test: the user sets p-value which is calculated from genotype frequencies in control samples. SNPs with a p-value below the settings are eliminated.

• Minimum call rate: the user sets the minimum call rate, which is calculated from "no call/call" ratio in all control samples, to avoid markers with lower call rates suggesting difficulties in genotyping.

• MAF zero test: markers where MAFs are zero can be eliminated.

• NoCall test (MLINK, Superlink): markers that are not called in any samples analyzed will be eliminated.

• Maximum confidence: confidence scores that are reliabilities of signal calling from hybridization can be set here. When the user skips this setting, the default value (for example 0.5 in BRLMM algorithm [16] as a default) defined in Genotyping Console™, which is Affymetrix genotyping software, will be used.

• Interval (Merlin, Allegro): minimum intermarker distances will be set. There are two marker-selecting methods, the min-max method and min MAF and interval method. In the min-max method, the user sets minimum and maximum intervals, then SNP with the highest MAF in the region defined by these intervals will be adopted. On the other hand, the min MAF and interval method select SNPs with MAFs higher than defined, and one SNP locating nearest to the minimum interval from the former SNP will be adopted.

• LD: the user sets the maximum D' and r^2 ^scores to eliminate neighboring markers in LD with D' or r^2 ^scores higher than the threshold. The reference LD data file containing all D' and r^2 ^data obtained from the Hapmap database [17] can be downloaded from our WEB sites. Information of four ethnic populations (CEU, CHB, JPT, and YRI) has been provided as LD data files thus far. Users can make LD data files from their own samples by using LD Data Maker in the Main Menu. Click on LD Data Maker and specify the directory where chip files located.

SNP HiTLink produces a binary file (.lkin file) containing the marker and pedigree information with parameter settings, and this file is transported from Windows OS to Unix OS. Perl programming (run_linkage.pl) performs MLINK, Superlink, Merlin or Allegro against a specified '.lkin' file. Whole genome analysis will be carried out automatically but the user can also specify a chromosome number by option when analyzing only the chromosome of interest. Outputs of haplotype prediction by Allegro in a specific text format are easily visualized on the windows system by using the haplotype viewer implemented in this system. Data are shown in columns and can be copied to an Excel sheet for further use [see also the manual of Additional file 4].

## Result and discussion

**Figure 4:**
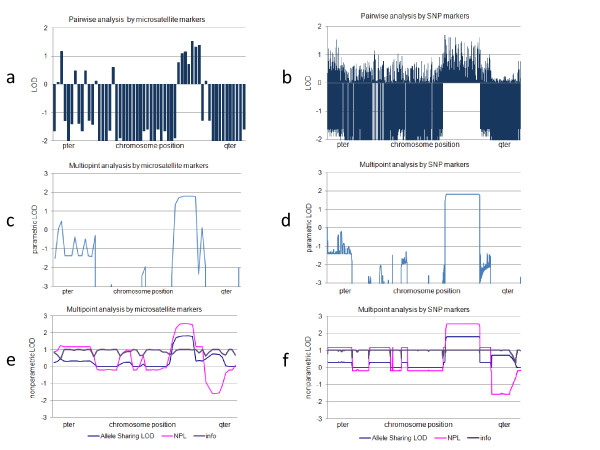
**Results of pairwise analysis (a and b) by MLINK, multipoint parametric analysis (c and d), and multipoint nonparametric analysis (e and f) by Allegro employing microsatellite (a, c and e) and 100KSNP (b, d, and f) markers**. SNP markers were selected as confidence score < 0.1, HWE > 0.05, call rate > 0.95, and intervals of 100 kb (for multipoint analysis). The x-axis represents the position on each chromosome and the y-axis represents calculated parametric LOD scores (allele sharing LOD), nonparametric linkage scores (NPL), or information measures (info).

Figure 4 shows results of pairwise and multipoint analysis of a pedigree using the Affymetrix Mapping 100 K array set along with results obtained using microsatellite (ABI PRISM^® ^Linkage Mapping Set) data. SNPs and microsatellite markers showed similar results in both pairwise and multipoint analyses but a higher resolution and a clearer border of regions where comparably high LOD scores were expected were achieved using SNP markers. These results indicated that SNP data were comparable or even superior to those obtained from microsatellite markers. The maximum LOD scores of pairwise analysis using microsatellite and SNP markers, were 1.7 and 1.5, respectively. In multipoint analysis, maximum parametric LOD score of 1.8, and nonparametric allele sharing LOD and NPL scores of 1.8 and 2.4, respectively, were obtained using both microsatellite and SNP markers.

**Figure 5:**
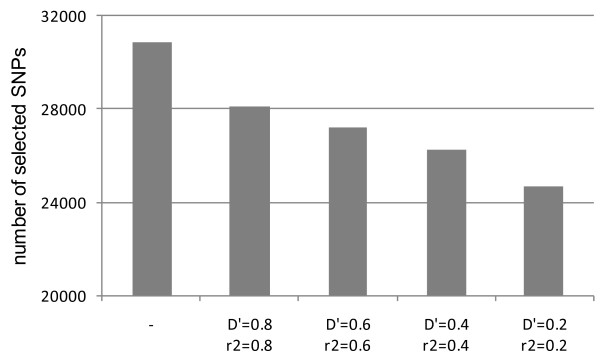
**Number of markers on chromosome 1 employed in multipoint analysis (intervals of 100–500 bp, confidence score < 0.02, and HWE > 0.05, call rate = 1) with varied LD settings**. DNA obtained from two affected siblings of a family was analyzed using Genome-Wide Human SNP array 6.0.

**Figure 6:**
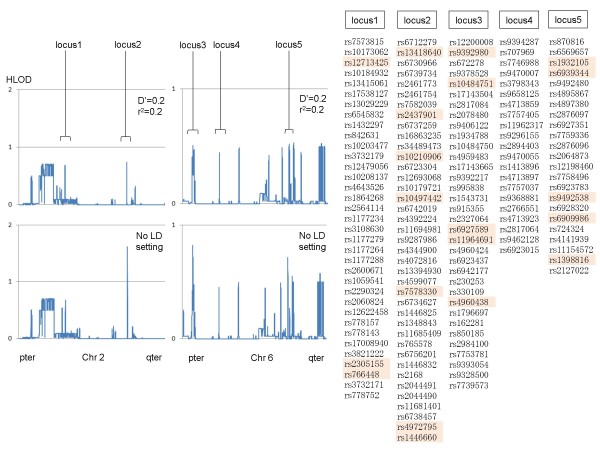
**Effect of LD between markers on multipoint parametric heterogeneity LOD scores on chromosome 2 and 6**. Multipoint analysis by Allegro (intervals of 100–500 bp, confidence score < 0.02, and HWE > 0.05) were conducted with strict LD settings (D' = r^2 ^= 0.2) or without settings. Results of chromosome 2 and 6 were shown. DNAs obtained from four affected sibling pairs were analyzed by Genome-Wide Human SNP array 6.0. SNP IDs of five loci were extracted. Colored SNP IDs are those eliminated in analysis with LD settings.

We tested the effect of LD setting on the number of markers and LOD scores of parametric multipoint analysis employing Genome-Wide Human SNP array 6.0. Approximately 70000 SNP markers are placed on chromosome 1 of SNP array 6.0. Of these, about 31000 were selected with parameter settings of 100–500 bp interval, call rate = 1, confidence score < 0.02, and HWE > 0.05. SNP markers were eliminated proportionately with decreasing D' and r^2 ^and about 28000 SNP markers were retained when D' = 0.2 and r^2 ^= 0.2, indicating that there are many neighboring markers that are in LD from each other (Figure 5). When multipoint parametric linkage analysis of four pedigrees including two affected siblings without parent genotypes was conducted without setting a LD threshold, the multipoint HLOD (heterogeneity LOD) scores showed inflation compared with those obtained at the setting of D' < 0.2, r^2 ^< 0.2 (Figure 6). Inflation was severer at the loci employing many markers in LD (loci 2, 3 and 5) than at the locus where no or only few LD markers were found (locus 1 and 4), suggesting this inflation was mainly due to the LD of markers. Given that our result was obtained from only four families with two affected siblings, markers in LD can have serious effects on the calculation of LOD scores when a large number of families are simultaneously analyzed, as sometimes LOD scores can inflate markedly as simulated in a previous study [10].

The runtime for preparing lkin files is less than 10 minutes (usually from about 10 second to a few minutes), and the runtime of whole genome linkage analysis of a pedigree performed using general personal computer was about 4 hours for pairwise analysis, when using all of approximately 1 million markers on Genome-Wide Human SNP array 6.0. For multipoint analysis less than 1 hour was required even in the case of a family including consanguineous loops when intermarker distances were set to be varied from 300 bp to 100 kbp. These results show that extremely dense markers that are now mainly utilized for the genome wide association study (GWAS) can also be utilized for high-throughput linkage analysis.

## Conclusion

We have developed the SNP HiTLink, system for executing parametric/nonparametric linkage analysis using SNP data. This is the first and unique system that directly accepts recent 100 K, 500 K and 1 M markers of Affymetrix SNP CHP files and prepares very flexible marker-selecting implementations for linkage analysis, although some convenient pipelines that pass the SNP data to a linkage analysis program [18,19] or tools for visualization and removal of LD [20,21] have been developed thus far. The results using this system were comparable or even superior to those obtained using microsatellite markers, convincing us the advantage of using SNP data obtained by DNA microarray for linkage analysis. The number of SNP data located on a single chip is continuing to increase owing to recent developed technologies and demands for dense markers for GWAS. On the other hand, we should be carefully concerned about typing error data when using such dense SNP data for multipoint linkage analysis. Quite flexible marker-selecting implementations on SNP HiTLink will be advantageous from this point of view. Although SNP HiTLink only accepts Affymetrix SNP Chip files, improvements that support multiple platforms for SNP typing such as Illumina are required in the future. Furthermore, more user-friendly interface where analyses can be processed simply (for instance, through integrated single GUI) rather than transporting files from Windows to Unix OS, will be desirable. This system can be widely applied for linkage analysis using microarray-based SNP data, with which one can expect high-throughput and reliable linkage analysis.

## Authors' contributions

YF, HA and EN dealt with the computational aspects in development of the system, and YF carried out analyses of the data. YN, YT performed SNP genotyping, and AM, RK performed microsatellite genotyping. HD, JG contributed to general planning and interpretation. ST provided overall guidance for this project. All authors read and approved the final manuscript.

## Supplementary Material

Additional file 1 **SNP HiTLink**. The SNP HiTLink main program. Click here for file

Additional file 2 **run_linkage.pl**. The run_linkage.pl program for executing linkage analysis program in Unix OS. Click here for file

Additional file 3 **sample_data**. Sample file set including a map file, pedin.dat and pedin.pre file. Click here for file

Additional file 4 **SNP HiTLink manual**. The manual for SNP HiTLink. Click here for file

## References

[B1] Evans DM, Cardon LR (2004). Guidelines for genotyping in genomewide linkage studies: single-nucleotide-polymorphism maps versus microsatellite maps. Am J Hum Genet.

[B2] Matise TC, Sachidanandam R, Clark AG, Kruglyak L, Wijsman E, Kakol J, Buyske S, Chui B, Cohen P, de Toma C (2003). A 3.9-centimorgan-resolution human single-nucleotide polymorphism linkage map and screening set. Am J Hum Genet.

[B3] John S, Shephard N, Liu G, Zeggini E, Cao M, Chen W, Vasavda N, Mills T, Barton A, Hinks A (2004). Whole-genome scan, in a complex disease, using 11,245 single-nucleotide polymorphisms: comparison with microsatellites. Am J Hum Genet.

[B4] Cottingham RW, Idury RM, Schaffer AA (1993). Faster sequential genetic linkage computations. Am J Hum Genet.

[B5] Lathrop GM, Lalouel JM, Julier C, Ott J (1984). Strategies for multilocus linkage analysis in humans. Proc Natl Acad Sci USA.

[B6] Montgomery GW, Campbell MJ, Dickson P, Herbert S, Siemering K, Ewen-White KR, Visscher PM, Martin NG (2005). Estimation of the rate of SNP genotyping errors from DNA extracted from different tissues. Twin Res Hum Genet.

[B7] Hong H, Su Z, Ge W, Shi L, Perkins R, Fang H, Xu J, Chen JJ, Han T, Kaput J (2008). Assessing batch effects of genotype calling algorithm BRLMM for the Affymetrix GeneChip Human Mapping 500 K array set using 270 HapMap samples. BMC Bioinformatics.

[B8] Saunders IW, Brohede J, Hannan GN (2007). Estimating genotyping error rates from Mendelian errors in SNP array genotypes and their impact on inference. Genomics.

[B9] Gordon D, Heath SC, Ott J (1999). True pedigree errors more frequent than apparent errors for single nucleotide polymorphisms. Hum Hered.

[B10] Huang Q, Shete S, Amos CI (2004). Ignoring linkage disequilibrium among tightly linked markers induces false-positive evidence of linkage for affected sib pair analysis. Am J Hum Genet.

[B11] Schaid DJ, McDonnell SK, Wang L, Cunningham JM, Thibodeau SN (2002). Caution on pedigree haplotype inference with software that assumes linkage equilibrium. Am J Hum Genet.

[B12] Fishelson M, Geiger D (2002). Exact genetic linkage computations for general pedigrees. Bioinformatics.

[B13] Abecasis GR, Cherny SS, Cookson WO, Cardon LR (2002). Merlin – rapid analysis of dense genetic maps using sparse gene flow trees. Nat Genet.

[B14] Gudbjartsson DF, Jonasson K, Frigge ML, Kong A (2000). Allegro, a new computer program for multipoint linkage analysis. Nat Genet.

[B15] Gudbjartsson DF, Thorvaldsson T, Kong A, Gunnarsson G, Ingolfsdottir A (2005). Allegro version 2. Nat Genet.

[B16] Affymetrix I (2006). BRLMM: an Improved Genotype Calling Method for the GeneChip^® ^Human Mapping 500 K Array Set. http://www.affymetrix.com/support/technical/whitepapers/brlmm_whitepaper.pdf.

[B17] The International HapMap Consortium (2003). The International HapMap Project. Nature.

[B18] Hoffmann K, Lindner TH (2005). easyLINKAGE-Plus – automated linkage analyses using large-scale SNP data. Bioinformatics.

[B19] Hiekkalinna T, Peltonen L (1999). New program: AUTOSCAN 1.0 automated use of linkage analysis programs. American Journal of Human Genetics.

[B20] Webb EL, Sellick GS, Houlston RS (2005). SNPLINK: multipoint linkage analysis of densely distributed SNP data incorporating automated linkage disequilibrium removal. Bioinformatics.

[B21] Gaunt TR, Rodriguez S, Zapata C, Day IN (2006). MIDAS: software for analysis and visualisation of interallelic disequilibrium between multiallelic markers. BMC Bioinformatics.

