# Supplementary material for: SNP HiTLink: a high-throughput linkage analysis system employing dense SNP data
Source: BMC Bioinformatics. 2009 Apr 24;10:121. doi: 10.1186/1471-2105-10-121 (PMC2680848; doi:10.1186/1471-2105-10-121)
Supplement: Additional file 4 — SNP HiTLink manual. The manual for SNP HiTLink. [file 1471-2105-10-121-S4.pdf]

SNP HiTLink Manual

Yoko Fukuda[1], Hiroki Adachi[2], Eiji Nakamura[2], and Shoji Tsuji[1]
[1] Department of Neurology, Graduate School of Medicine, the University of Tokyo, Tokyo, Japan
[2] Dynacom Co., Ltd, Kanagawa, Japan

Last updated; 19 Mar 2009

General notes

SNP HiTLink (SNP High-Throughput Linkage Analysis System) is a computer program providing a useful pipeline to directly connect single nucleotide polymorphism (SNP) data and linkage analysis program. SNP data are now widely recognized as powerful markers for linkage analysis. To apply SNP data to genomewide high-throughput linkage analysis, however, requires several complicated steps such as elimination of typing error data, markers in linkage disequilibrium (LD) and preparation of linkage format files containing selected SNP information.

SNP HiTLink currently supports the Affymetrix Mapping 100k/500k array set and Genome-Wide Human SNP array 5.0/6.0 and can carry out typical linkage analysis programs of MLINK (FASTLINK/ LINKAGE package)[1, 2], Superlink[3], Merlin[4] and Allegro[5,6]. Pairwise linkage analysis can be performed by MLINK, Superlink and Allegro. Merlin and Allegro provides the multipoint parametric/non-parametric linkage analysis. SNP HiTLink consists of two processes. The first process creates necessary data files by the program described in the Visual Basic programming on Windows OS, and these files are then transferred to Unix OS. The Perl script files invoke necessary linkage programs with necessary data files on Unix OS.

Installation

To install the SNP HiTLink, download the Windows installer package from Dynacom web site (https://www.dynacom.co.jp/u-tokyo.ac.jp/snphitlink). Expand “SNP HiTLink Installer” and double-click “SNP HiTLink Setup” icon.

Execution

The SNP HiTLink requires Windows XP SP2 or later versions/ Vista (32Bit) and unix (supporting perl5) OS. Mlink, Superlink, Merlin and Allegro should be installed in unix OS. MLINK, included in the FASTLINK package, can be downloaded from ftp://fastlink.nih.gov/pub/fastlink. Superlink and Merlin are available from http://cbl-fog.cs.technion.ac.il/superlink/ and http://www.sph.umich.edu/csg/abecasis/Merlin/download/, respectively. Allegro is available from deCODE genetics, Inc.

Double-click “SNP HiTLink” to begin the program.

Preparation for linkage analysis

1. Annotation files

   Annotation files containing all information of SNPs such as general IDs and chromosomal positions can be obtained from the Affymetrix web page (https://www.affymetrix.com/support/technical/annotationfilesmain.affx). Choose annotation files corresponding to the SNP chip the users select, and download them. The Annotation File Manager on the Main Menu registers each of the annotation files to recognize them in linkage analysis. When using a mapping 100K or 500K array, register both Mapping50K_Hind and Mapping50K_Xba or Mapping250K_NSP and Mapping250K_Sty, respectively.

2. Allele frequency files

   From CHP files of control samples, allele frequency can be automatically calculated by the Allele Frequency Data Maker. Click on Allele Frequency Data Maker, and specify the directory where CHP files are located, then choose the array type and enter the title name. Clicking on Make icon will create allele frequency data.

3. LD data files

   LD data files can be downloaded from our web site (https://www.dynacom.co.jp/adachi/linkage/data/hapmap). These files contain all the data of D' and r^2 of four ethnic populations available from the hapmap database. Download LD data files of interest and save them in an appropriate directory. Users can make LD data files from their own samples by using LD Data Maker in the Main Menu. Click on LD Data Maker and specify the directory where chip files located.

Input files

1. CHP files

CHP files are generated by Affymetrix Genotyping Console ^{Tm} from firstly created CEL files in genotyping assays. It is preferable that CHP files employed in the same linkage analysis are saved in the same directory. Names of CHP files should be started with an identical name (e.g., aaa_SNP6.chp/bbb_SNP6.chp etc.). Do not start with common letters such as the version of the SNP chip or date of assay (e.g., SNP6_aaa.chp/SNP6_bbb.chp).

2. Pedin.dat (without genotype information)

Pedin.dat is a text file for MLINK and Superlink that contains pedigree data described by the linkage file format. Make pedin.dat using a general text editor, and type '0 0' in the column for genotype information. See manuals of each program for detailed description of consanguineous marriages and probands.

3. Pedin.pre (without genotype information)

Pedin.pre is a text file similar to pedin.dat but used in analysis of Merlin and Allegro. Type '0 0' in the column for genotype information.

4. CHP Mapping files

Mapping file (map.txt) is a tab-separated text file used to connect a person's ID number in pedin.dat or pedin.pre to an individual in CHP files. Mapping file contains the names of the CHP files, PedIds, and PerIds. The lengths of the 'name' in the first column are preferably the same, as the program recognizes names from the head. If 'C10' is listed next to 'C1', 'C10' cannot be appropriately recognized because 'C10' also contains 'C1'.

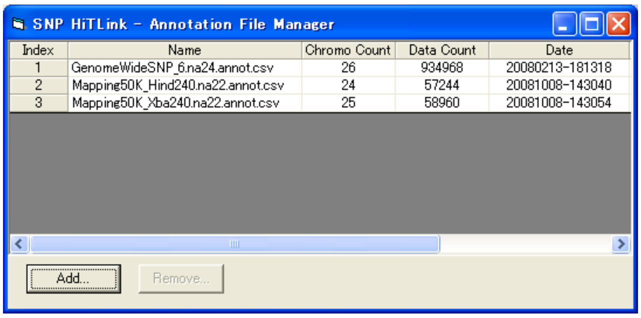

Annotation file Manager

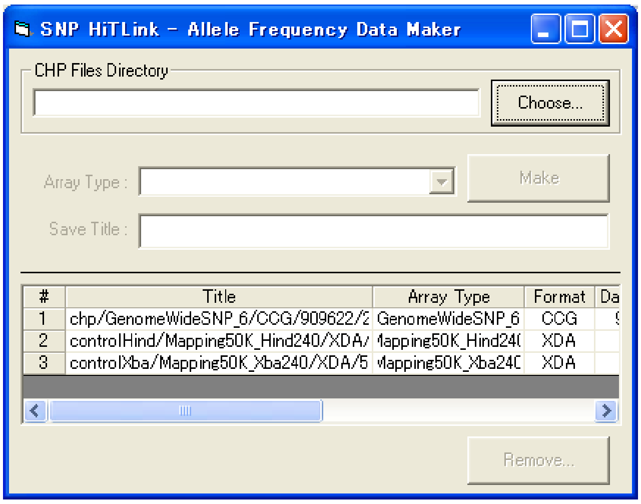

Allele Frequency Data Maker

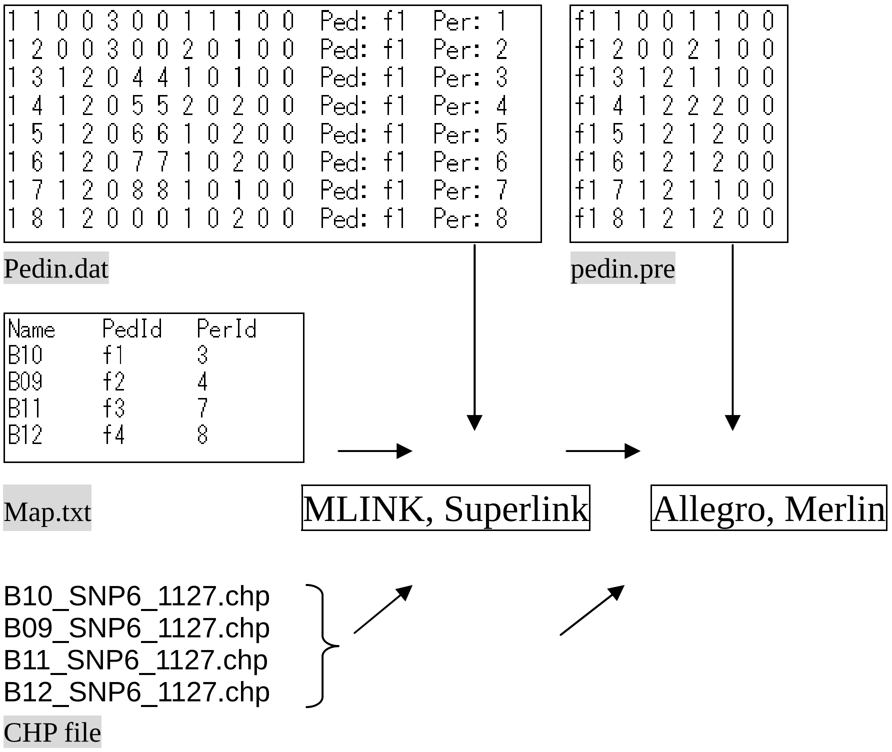

Linkage analysis - Build lkin file

Click on ‘Make lkin Data’ to create ‘lkin file’, that is a batch file for high-throughput linkage analysis. Choose an analysis program (MLINK, Superlink, Merlin or Allegro), pedin.dat/pedin.pre, a CHP Mapping file and a CHP file directory. Disease gene frequencies, liability class, and penetrances should be set as usual linkage analysis. When using more than one liability classes (not supported for Merlin), add a column of liability class in pedin.dat or pedin.pre. Merlin and Allegro MODEL can be selected in the same manner in the original program. Click on ‘Edit’ to change, add and remove a model. Refer to the manual of Merlin and Allegro for further details on MODEL.

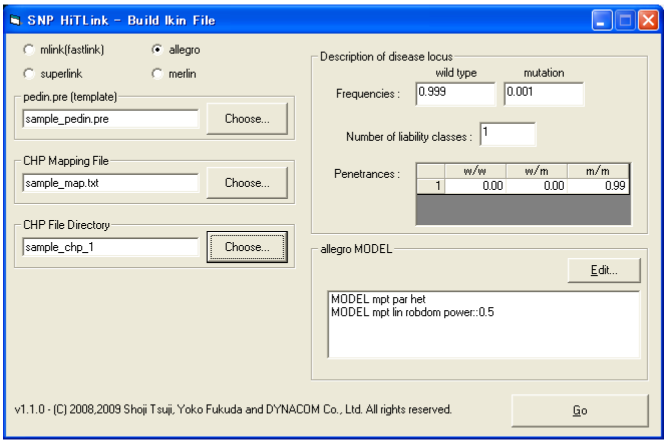

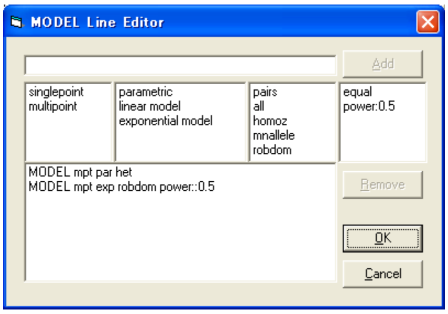

CHP File settings

Specify 'Annotation File' and 'Allele Frequency' registered by 'Annotation File Manager' and 'Allele Frequency Data Maker'. Recognized CHP files and the corresponding person's ID number in pedfile will be shown.

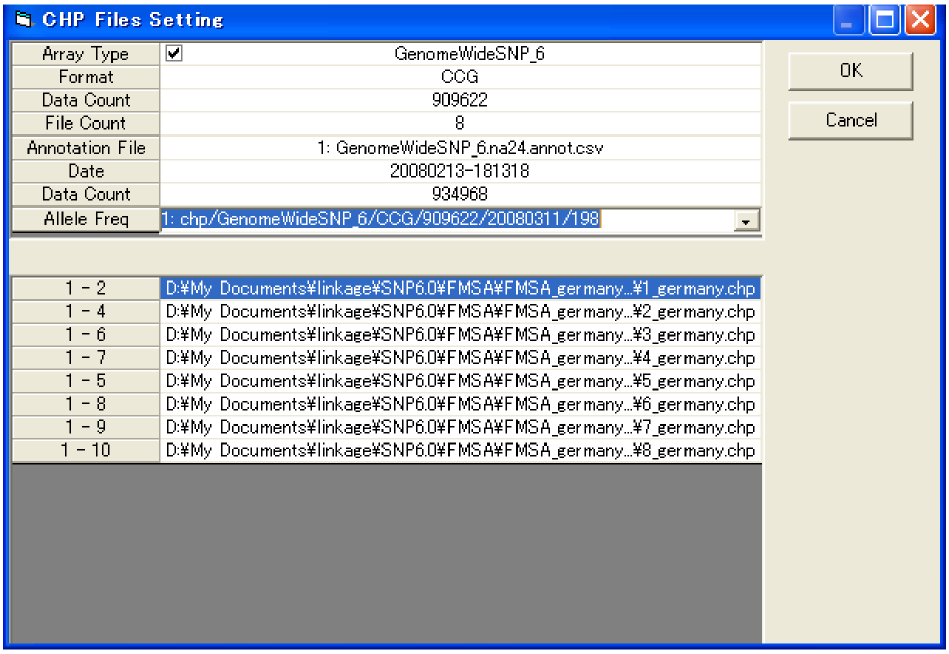

Option settings

SNP exclude settings

1. Minimum HWE-test p-value

P-value is calculated from genotype frequencies in control samples. SNPs with p-value below the settings are eliminated.

2. Minimum call rate

Call rate is calculated from "no call / call" ratio in all control samples to avoid markers with lower call rates suggesting difficulties in genotyping. Typically 0.9, 0.95, or 0.99 are used.

3. MAF zero test

Markers where minor allele frequencies (MAFs) are zero can be eliminated.

4. NoCall test (MLINK and Superlink)

Markers that are not called in any samples analyzed will be eliminated.

5. Maximum confidence

Confidence scores that are reliabilities of signal calling from hybridization can be set here. When users skips this setting, the default value (for example 0.5 in BRLMM algorithm as a default) defined in Genotyping Console^{TM}, which is Affymetrix genotyping software, will be used.

Interval settings (Merlin and Allegro)

Choose methods for setting inter-marker distances.

1. Min-max method

The minimum interval and maximum interval are set, among SNPs in the region defined by these intervals, one with the highest MAF is selected.

2. Min MAF & interval method

The minimum interval and minimum MAF are set. After filtering SNPs defined as having higher MAFs, one SNP longer than the minimum interval from the former SNP is selected.

Use LD settings

LD data files are available from our download site, or users can make from their own samples. Users set parameters of LD by setting D' and r^2. By referring to the LD Data file specified by users, the program constructs 'LD blocks' where neighboring SNPs with D' or r^2 higher than defined are included in the same blocks. When a marker A is in the same LD block as the former markers, the program skips marker A and goes to the next marker. Click on 'Test' to see information on the LD block defined by D' and r^2.

Do haplotyping setting (Allegro)

Haplotype prediction originally implemented in Allegro is carried out by checking on this box.

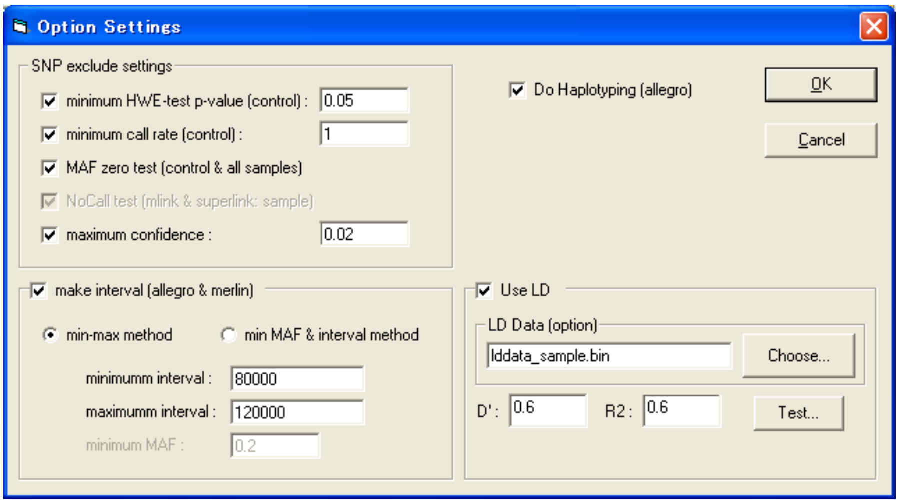

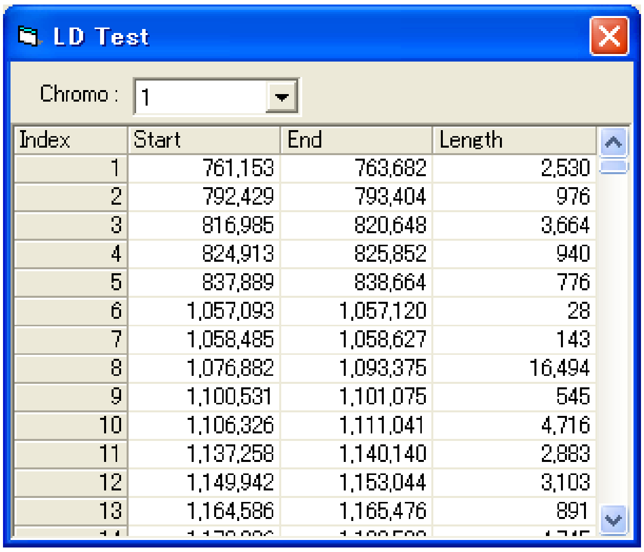

Index	Start	End	Length
1	761,153	763,682	2,530
2	792,429	793,404	976
3	816,985	820,648	3,664
4	824,913	825,852	940
5	837,889	838,664	776
6	1,057,093	1,057,120	28
7	1,058,485	1,058,627	143
8	1,076,882	1,093,375	16,494
9	1,100,531	1,101,075	545
10	1,106,326	1,111,041	4,716
11	1,137,258	1,140,140	2,883
12	1,149,942	1,153,044	3,103
13	1,164,586	1,165,476	891
[illegible]	[illegible]	[illegible]	[illegible]

Transfer of lkin files to unix OS

Transfer lkin files to unix OS by binary mode.

Running linkage program

A perl program, run_linkage.pl available from the Dynacom Website should be transferred to unix and decompressed. To decompress, type the following.

%gunzip run_linkage_xxx.tgz

% tar xvf run_linkage_xxx.tar

To run linkage program, type

% ./run_linkage.pl fileneme.lkin

run_linkage.pl automatically executes MLINK, Superlink, Merlin or Allegro by recognizing the lkin file format. In the analysis using MLINK and Superlink, output_xx.txt files are output files separated by chromosomes. Information on skipped markers is listed in skipped_xx.txt. In the analysis using Merlin and Allegro, a zip file containing directories of each chromosome is produced. To decompress, type %unzip xxx.zip

In each of the chromosome directories, users find output files of analyses that you specified in MODEL option.

When users check on ‘Do haplotype’ option in Allegro, haplo.out, founder.out, ihaplo.out, inher.out will be produced in each of the chromosome directories. The haplotype viewer included in the SNP HiTLink package visualizes those files in the table format that can be easily copied to an Excel sheet to be analyzed.

When users analyze a family with parental data, errors due to inconsistency between parents and

children may occur. Multipoint analysis by Allegro is usually interrupted by inconsistent genotypes. SNP HiTLink has the functionality to detect and skip those SNPs in multipoint analysis by referring to 'unknown' (mlink) or 'superlink' (superlink) errors described in output_xx.txt files produced by pairwise analysis. Pair-wised analysis should precede multipoint analysis by Allegro to effectively skip inconsistent markers. Run_linkage.pl of lkin files for multipoint analysis should be run in the same directory where output_xx.txt files are saved.

References

1. Cottingham RW Jr, Idury RM, Schaffer AA, *Faster sequential genetic linkage computations.* Am J Hum Genet, 1993. **53**(1): p. 252-63.
2. Lathrop GM et al., *Strategies for multilocus linkage analysis in humans.* Proc Natl Acad Sci U S A, 1984. **81**(11): p.3443-6.
3. Fishelson M, Geiger D, *Exact genetic linkage computations for general pedigrees.* Bioinformatics. 2002. **18** Suppl 1:S189-98.
4. Abecasis GR, Cherny SS, Cookson WO, Cardon LR, *Merlin--rapid analysis of dense genetic maps using sparse gene flow trees.* Nat Genet. 2002. **30**:p.97-101.
5. Gudbjartsson DF et al., *Allegro, a new computer program for multipoint linkage analysis.* Nat Genet, 2000. **25**: p. 12-3.
6. Gudbjartsson, D.F., et al., *Allegro version 2.* Nat Genet, 2005. **37**(10): p. 1015-6.
